# Ruth vs. Reuling

**Published:** 1880-07

**Authors:** 


"RUTH VS. REULING," is the caption of a scurrilous editorial in the June, ultimo, issue of "The Southern Clinic," against the moral and professional reputation of a prominent medical gentleman of this city. We feel it incumbent, as independent journalists, and fair dealing conservators of professional character and reputation, to lay before our readers the action of the committee appointed by the Medico-Chirurgical Faculty of this city, our State Society, to investigate statements involving Dr. Chisolm's connection with the suit of *Ruth vs. Reuling*. Indeed, we should feel derelict in duty, as well as lacking in a proper appreciation of *justice* to the professional character and reputation of a fellow citizen, were we to pass in silence even, the *ex-parte* and malevolent words and innuendo of the editor of "Clinic." The following are the unanimous resolutions of the committee, after careful examination of witnesses, and as they are a complete refutation of the reprehensible language of the editorial referred to, we will introduce them without further comment, viz.: "1st. That *Dr. Chisolm is entirely innocent of the charges made against him by Dr. Reuling.* 2d. That *Dr. Reuling had not the slightest foundation* for his accusation against Dr. Chisolm. 3d. *That the evidence undoubtedly shows* that *so far from instigating the suit against Dr. Reuling, he advised the Ruths and their counsel, not to bring suit.*" These resolutions, we are informed, were unanimously accepted by the State Society.

